# Artificial intelligence may offer insight into factors determining individual TSH level

**DOI:** 10.1371/journal.pone.0233336

**Published:** 2020-05-20

**Authors:** Prasanna Santhanam, Tanmay Nath, Faiz Khan Mohammad, Rexford S. Ahima

**Affiliations:** 1 Division of Endocrinology, Diabetes, & Metabolism, Department of Medicine, Johns Hopkins University School of Medicine, Baltimore, Maryland, United States of America; 2 Department of Biostatistics, Johns Hopkins University School of Medicine, Baltimore, Maryland, United States of America; 3 Department of Chemical Engineering, Indian Institute of Technology, Madras, India; Wroclaw University of Science and Technology, POLAND

## Abstract

The factors that determine Serum Thyrotropin (TSH) levels have been examined through different methods, using different covariates. However, the use of machine learning methods has so far not been studied in population databases like NHANES (National Health and Nutritional Examination Survey) to predict TSH. In this study, we performed a comparative analysis of different machine learning methods like Linear regression, Random forest, Support vector machine, multilayer perceptron and stacking regression to predict TSH and classify individuals with normal, low and high TSH levels. We considered Free T4, Anti-TPO antibodies, T3, Body Mass Index (BMI), Age and Ethnicity as the predictor variables. A total of 9818 subjects were included in this comparative analysis. We used coefficient of determination (r^2^) value to compare the results for predicting the TSH and show that the Random Forest, Gradient Boosting and Stacking Regression perform equally well in predicting TSH and achieve the highest r^2^ value = 0.13, with mean absolute error of 0.78. Moreover, we found that Anti-TPO is the most important feature in predicting TSH followed by Age, BMI, T3 and Free-T4 for the regression analysis. While classifying TSH into normal, high or low levels, our comparative analysis also shows that Random forest performs the best in the classification study, performed with individuals with normal, high and low levels of TSH. We found the following Areas Under Curve (AUC); for low TSH, AUC = 0.61, normal TSH, AUC = 0.61 and elevated TSH AUC = 0.69. Additionally, we found that Anti-TPO was the most important feature in classifying TSH. In this study, we suggest that artificial intelligence and machine learning methods might offer an insight into the complex hypothalamic-pituitary -thyroid axis and may be an invaluable tool that guides us in making appropriate therapeutic decisions (thyroid hormone dosing) for the individual patient.

## Introduction

TSH (Thyroid Stimulating Hormone, also called Thyrotropin) is secreted by the pituitary gland to stimulate the production of thyroid hormone by the thyroid. Primary hypothyroidism (approximately 99% of the cases) is characterized by an elevated TSH level while secondary hypothyroidism is due to lack of stimulation of a normal thyroid gland, as result of TSH deficiency from hypothalamic or pituitary disease[1]. TSH is the main target of thyroid hormone replacement in primary hypothyroidism [2]. The goal of hypothyroidism treatment is, to relieve the symptoms of hypothyroidism and achieve normalization of TSH levels and thyroid hormones[2]. Normal TSH based on epidemiological data, ranges widely between 0.4 and 4.0 and within this range, there is substantial variation in the population with respect to the TSH levels[2]. Clinicians often find it challenging to alleviate the symptoms of hypothyroidism and target the TSH at the appropriate level simultaneously. Moreover, each individual appears to have a predetermined optimal personal TSH level(may be genetically individualized) that is often unknowable, once primary hypothyroidism has developed as a clinical condition, and variations in assays, concurrent illness etc make it hard to achieve the right TSH level for the individual patient [3].

The factors that determine serum TSH levels have been examined through different methods, using different covariates. In a cohort of over 4000 participants from the Busselton Health Study, it was shown that logarithmic transformed TSH was related to free T4 in a complex, nonlinear way, and was influenced by age, smoking status, and the presence of Anti-TPO (Thyroperoxidase) antibodies [4]. Others have suggested that the relationship could be fourth- order polynomial, with gender and smoking both influencing the results [5]. In an earlier epidemiological study using NHANES (The National Health and Nutritional Examination Survey) III population-based database, higher TSH and the prevalence of anti-thyroid antibodies was more likely in females and elderly, with a higher prevalence in Whites and Mexican Americans[6]. African- Americans had a lower TSH and lower prevalence of thyroid autoantibodies[6].

Different machine learning methods have been used in recent times in health care settings, especially in the predictive analytics of high blood pressure, and diabetes [7]. As early as 1993, Artificial Neural Network was first used to assess thyroid function from in-vitro laboratory tests[8]. Since then, neural network has been used to distinguish between benign and malignant thyroid nodules using a feed- forward architecture[9]. The capability of AI methods to predict TSH from most commonly measured laboratory parameters and collected demographic information is largely unknown. We performed a comparative analysis of different machine learning methods. The aim of the research was to explore the potential of artificial intelligence for understanding the determinants of TSH based on usually obtained demographic information and laboratory parameters.

## Materials and methods

This was a retrospective study done after obtaining publicly available data from the CDC (https://wwwn.cdc.gov/nchs/nhanes/Default.aspx). The data had been collected after NCHS research ERB (Ethics Board Review) approval and we obtained local IRB exempt status after expedited review. The NHANES publishes continuous data from 1999–2000 annually. The continuously obtained data from 2007–2012 was compiled analysed [10]. The household questionnaire and phlebotomy files were linked to the laboratory data file using the unique survey participant identifier SEQN (Sequence) as per the analysis guidelines (https://wwwn.cdc.gov/nchs/nhanes/analyticguidelines.aspx). NHANES encourages combining multiple years for a large analytical sample.

The thyroid function tests that have been measured in the NHANES include total and free thyroxine (ft4), total and free Triiodothyronine (T3) and TSH. The Access hypersensitive human thyroid-stimulating hormone (h TSH) assay, a 3^rd^ generation, two-site immunoenzymatic (“sandwich”) assay performed by the Collaborative Laboratory Services Ottumwa, Iowa has been used in the NHANES study population. Total T3 is a competitive immunobinding assay while the free T4 is a two–step enzyme immunoassay [10]. Demographic variables with respect to age (years at the time of screening), gender, ethnicity (White, African-American, Hispanic, Mexican and Other) as well as anthropometric measures (weight (in kilograms), BMI (kg/m^2^)) were tabulated.

### Data pre-processing

The first step in the data pre-processing involved techniques to represent the raw patient records in a structured data frame that could be easily fed into the machine learning models. During this step, the raw data was converted into a pandas data frame, where the column names represented the features and each row represent a patient record. Any errors in the datatype of the raw dataset was corrected in this step. The next step during the data pre-processing was to check for any missing values and outliers in the features.

There were patient records with missing values. The entire patient record was excluded from the analysis in case, there were any missing values found in the features. Based on the answered questionnaire as well as medication review, persons with history of thyroid disorders were removed from the study. Levothyroxine treatment might lead to lowering of TSH levels (causing exogenous thyrotoxicosis) especially if the dose is not appropriate [2].

Participants with TSH greater than 10 and less than 0.1 were also removed from the study cohort since they represented overt hypothyroidism and hyperthyroidism respectively.

There were initially 11638 individuals out of which 9818 cases were selected for the machine learning analysis (Excluded due to thyroid condition or missing values: n = 1674, outliers TSH > = 10.0: n = 65, TSH < 0.1: n = 81).

### Data analysis

Prior studies have shown that TSH variations are associated with age, sex, race and gender[11].

The study considered the following predictors: Free T4, Anti-TPO antibodies, T3, Gender, BMI, Age and Ethnicity. For the Classification analysis, the TSH (μIU/ml) was classified into: 1) mildly suppressed or low TSH (<0.4), 2) Normal TSH (0.4–4.5) and 3) mildly elevated or high TSH (>4.5). The TSH was analysed both as a continuous as well as categorical variable with the following cofactors- gender (male, female) and ethnicity (Mexican American, Hispanic, White, African American, Other) and the dependent covariates- Free T4(ng/dl), Total T3(ng/dl), TPO(IU/ml), age (years at screening), and BMI (Body Mass Index) (kg/m^2^).

For the supervised machine learning models to learn these different categories, these class labels were represented in the data frame as 0,1, and 2 for mild supressed, normal and elevated levels of TSH respectively. All the categorical variables in the data frame like gender and ethnicity are converted into dummy or indicator variables. All the features are normalized using the following standardization model: $X^{\prime }=\frac{X-\mu }{\sigma }$ where X is the original feature vector, *μ* is the mean of the feature vector and *σ* is its standard deviation.

In this study, we used the supervised machine learning models; Linear regression, Random forest, Gradient boosting, Support vector machine, Multi-layer perceptron and Stacked regression for predicting TSH (regression models). As part of the classification analysis, the patients were classified into one of the three classes i.e. normal, mildly suppressed and elevated levels of TSH (classification models). For either models, the entire dataset is split into 70% training and 30% testing dataset.

We found that in the different classification models, the training dataset was highly imbalanced where 94.82% of the training dataset had normal level of TSH, 1.95% of the training dataset had mildly suppressed level of TSH while 3.23% of the training dataset had an elevated level of TSH. Training a machine learning model on such disproportionate ratio of observations in different classes yields biased results towards majority class and poor classification in the minority classes. Therefore, we used Synthetic Minority Oversampling Technique for Nominal and Continuous (SMOTE-NC), a widely used technique for balancing the observations only in training dataset and not in the testing dataset[12].

We tuned the hyperparameters for each of the machine learning models, except for Linear regression and Stacked regression, using 5-fold cross validation grid search, a widely used technique that exhaustively searches for the best parameters. In order to avoid overfitting, we evaluated each of the models using 5-fold cross-validation strategy, where the training dataset is split into k smaller sets, in this case, the k = 5. Thereafter, the model was trained on k-1 folds and validated on the remaining part of the dataset. We computed the performance of k fold cross-validation for regression and classification models. Thereafter, we tested the tuned model on the testing dataset and used the coefficient of determination (r^2^) for assessing the accuracy of the predictive models and confusion matrix, ROC curves and F1-scores to determine the accuracy of the different classification models. We also determined the important features, that are helpful in regression and classification models and validated our results by using the 5-fold cross validation strategy. Tables 1 and 2 shows the key parameters used for each of the model for regression and classification models respectively. For Multi-layer Perceptron, the weight initialization was done through normalized initialization (also termed Xavier Initialization)[13].

**Table 1:** Key parameters of various machine learning models used for regression task.

Model	Parameters
Random forest	number of trees = 800, max depth of the tree = 10, minimum number of samples to be a leaf node = 4
Gradient boosting	number of boosting stages = 1000, max depth = 2, minimum number of samples required to be at leaf node = 3, learning rate = 0.01
Linear regression	calculate intercept = True
Support vector regression	regularization parameter = 10, gamma = 0.01, kernel = radial basis function
Multi-layer perceptron	hidden layers = 1, number of neurons = 50, activation function = rectified linear unit, solver for weight optimization = Adam, maximum iterations = 1000, weight initialization strategy = normalized initialization (Xavier initialization)
Stacking regression	final estimator = Ridge regression

**Table 2:** Key parameters of various machine learning models used for classification task.

Model	Parameters
Random forest	number of trees = 200, max depth of the tree = 80, minimum number of samples to be a leaf node = 3
Gradient boosting	number of boosting stages = 1250, max depth = 4, minimum number of samples required to be at leaf node = 5, learning rate = 0.1
Logistic regression	regularization = 1, penalty = L2
Support vector classification	regularization parameter = 100, gamma = 0.9, kernel = radial basis function
Multi-layer perceptron	hidden layers = 1, number of neurons = 50, activation function = rectified linear unit, solver for weight optimization = Adam, maximum iterations = 5000, weight initialization strategy = normalized initialization (Xavier initialization)
Stacking classifier	final estimator = Logistic regression

Our open source analysis was conducted in python version 3.6 (https://www.python.org) using the library Scikit Learn, pandas, numpy, seaborn, matplotlib[12, 14–18]. The open source codes for the analysis are attached in the supplementary material.

## Results

The results are outlined below in tables as well as figures.

The baseline characteristics are shown in Table 3.

**Table 3:** Characteristics of the study population.

	N	Mean	Standard deviation	Median	Skewness	Range
**Free T4(ng/dl)**	9818	0.80	0.14	0.80	1.44	3.22
**TSH(μIU/ml)**	9818	1.81	1.17	1.52	1.99	9.67
**Anti-TPO(IU/ml)**	9818	16.20	79.86	0.60	7.31	1014.6
**T3(ng/dl)**	9818	116.70	25.34	114.00	2.23	670
**Weight(kg)**	9818	78.60	21.27	75.80	1.00	194.2
**BMI (kg/m**^**2**^**)**	9818	28.01	6.69	27.10	1.07	60.25

49.04% of the participants were women. The frequency distribution for the ethnicity was as follows; Whites—42.92% African-American-20.90%, Hispanic 11.31%, presence of Mexican origin 17.63%, and others 7.24%. We used 5-fold cross-validation on the training dataset. Table 4 shows the performance of 5-fold cross validation (to access the performance of the model and mitigate overfitting) of predictive models. We used coefficient of determination (r^2^) and mean absolute error to compare the predictive models. Table 5 shows the performance of 5-fold cross-validation of classification models. We used precision, recall and F1 measure for each class i.e. low, normal and elevated level of TSH to assess the performance of classification models.

**Table 4:** Performance of 5-fold cross-validation of predictive models (training data).

Model	R^2^ (Coefficient of determination)	MAE (Mean Absolute Error)
Random Forest	0.09 (0.01)	0.68
Gradient Boosting	0.10 (0.01)	0.68
Linear Regression	0.08 (0.01)	0.68
Support Vector Regression	0.06 (0.01)	0.65
Multi-layer Perceptron	0.08 (0.02)	0.68
Stacking Regression	0.10 (0.01)	0.67

Results in Mean ± SD. The Mean Absolute errors had the same standard deviation of 0.02.

Cross validation results show that none of the machine learning models overfit on the data.

**Table 5:** Performance of 5-fold cross-validation of classification models -training data (mean ± SD).

Model	Low			Normal			Elevated		
	Precision	Recall	F1	Precision	Recall	F1	Precision	Recall	F1
Random forest	0.95±0.02	0.95±0.02	0.95±0.02	0.93±0.04	0.87±0.01	0.9±0.02	0.92±0.05	0.93±0.01	0.92±0.03
Gradient boosting	0.95±0.01	0.96±0.02	0.96±0.01	0.95±0.07	0.96±0.01	0.95±0.04	0.92±0.01	0.94±0.04	0.93±0.02
Logistic regression	0.62±0.03	0.44±0.06	0.52±0.05	0.57±0.08	0.4±0.06	0.45±0.03	0.61±0.06	0.49±0.01	0.54±0.02
Support vector Classifier	0.93±0.03	0.97±0.01	0.95±0.02	0.93±0.03	0.84±0.01	0.88±0.01	0.9±0.06	0.95±0.01	0.93±0.04
Multi-layer perceptron	0.87±0.04	0.91±0.02	0.89±0.02	0.85±0.08	0.73±0.03	0.77±0.01	0.82±0.08	0.83±0.03	0.83±0.03
Stacking Classifier	0.97±0.01	0.97±0.02	0.97±0.01	0.96±0.07	0.96±0.01	0.96±0.03	0.96±0.03	0.95±0.03	0.95±0.02

(values in decimals mean %, for e.g. 0.95 implies 95%).

Cross validation results show that none of the machine learning models overfit on the data.

### Results of the regression analysis

**Fig 1:**
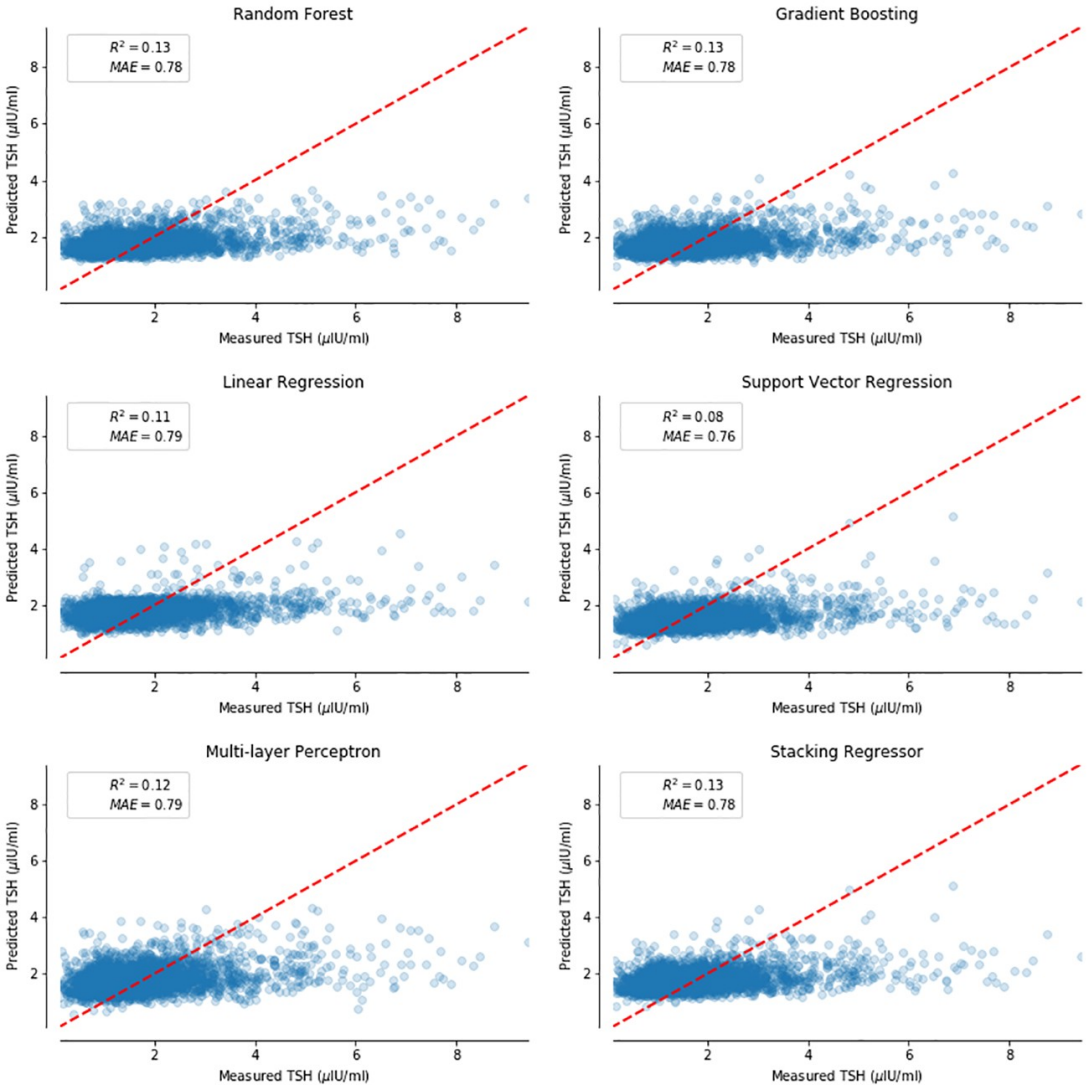
Parity plots- figure shows the comparison of measured against predicted TSH value for different machine learning models. The models are compared using the coefficient of determination (r^2^) and Mean absolute error (MAE). Each point in a parity plot represents a data point from testing dataset. A red line is added as a reference which indicates if the measured value is equal to predicted value.

**Fig 2:**
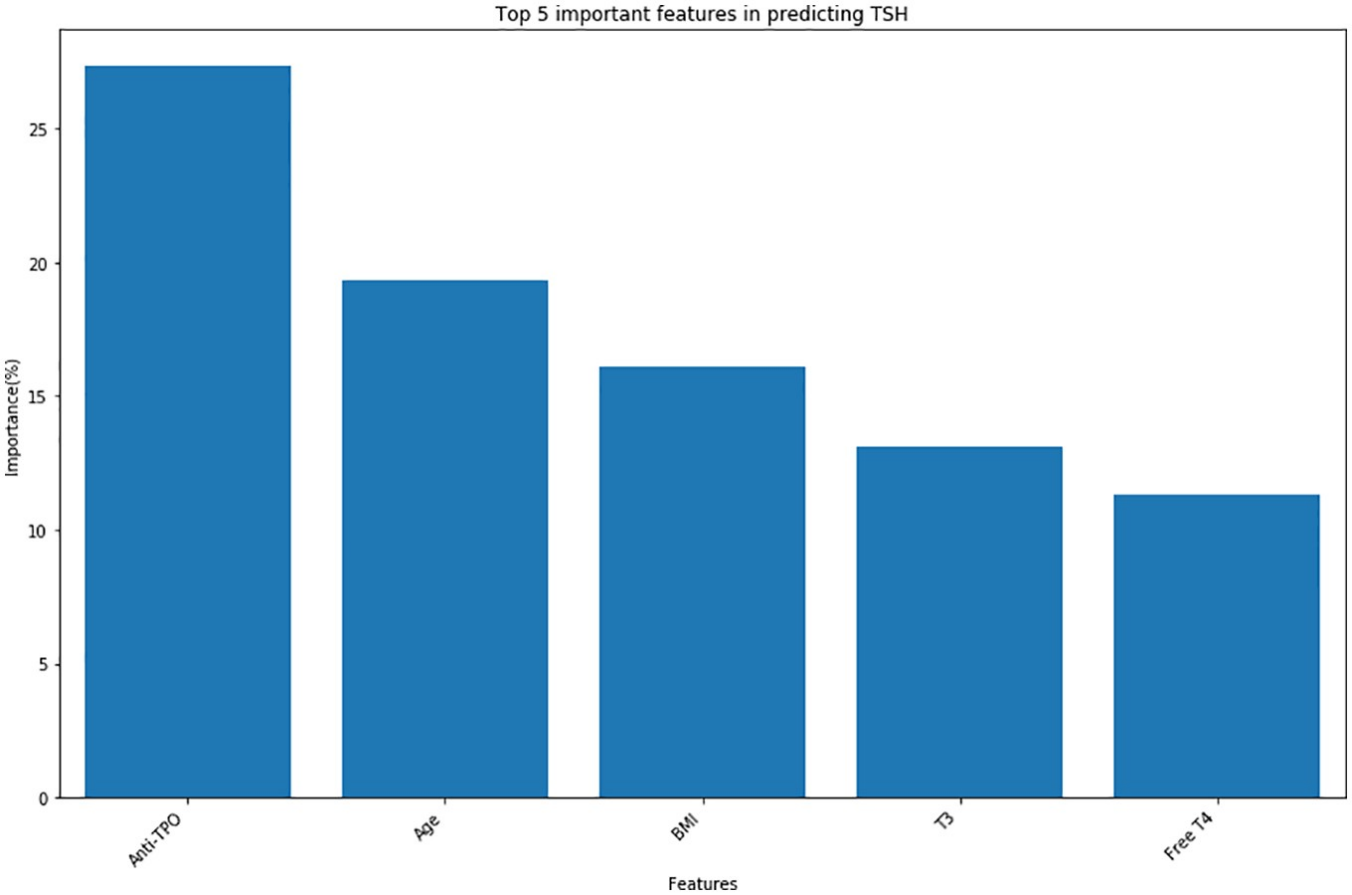
Feature importance for regression task—This shows the top 5 important features for predicting TSH using random forest in a testing dataset. We compute a score for all the features which indicates how useful it is in the construction of decision tree using Random forest. The top 5 variables in the x-axis are shown against their scores in y-axis.

We tested the tuned predictive and classification models on the testing dataset. “Fig 1” shows the parity plot for each of the predictive models. The parity plot compares the measured TSH value with the predicted TSH value. The results show that the Random Forest, Gradient Boosting and Stacking Regression perform equally well in predicting TSH. When Random Forest was used to compute the feature importance to predict TSH, Anti-TPO was the key determinant. “Fig 2” shows that Anti-TPO is the most important feature in predicting TSH followed by Age, BMI, T3 and Free-T4.

### Results of the classification analysis

Altogether, Random Forest achieves better performance compared to other models and achieves an Area Under Curve (AUC) = 0.61 for low, 0.61 for normal and 0.69 for elevated level of TSH. Random Forest also achieves the highest F1-measure for classifying the elevated level of TSH.

Figs “3” and “4” show the confusion matrices and Receiver Operating Characteristics (ROC) curve for each of the machine learning model on the testing dataset and outlines the relative superiority of Random Forest Method. We used Random Forest to compute the features that are important to classify TSH.

**Fig 3:**
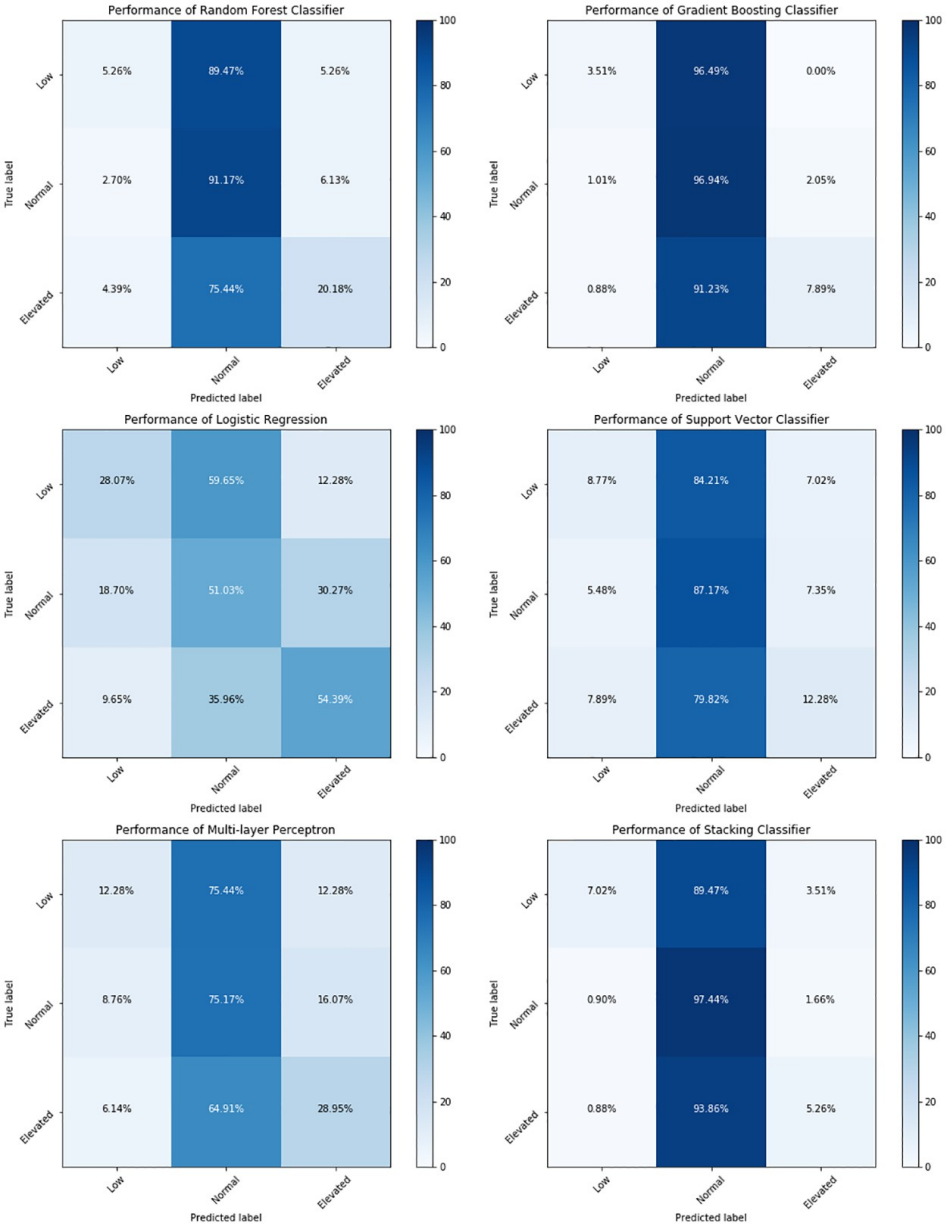
Confusion matrices—It shows the comparison of different machine learning models in classifying low, normal and elevated levels of TSH in a testing dataset using a confusion matrix.

**Fig 4:**
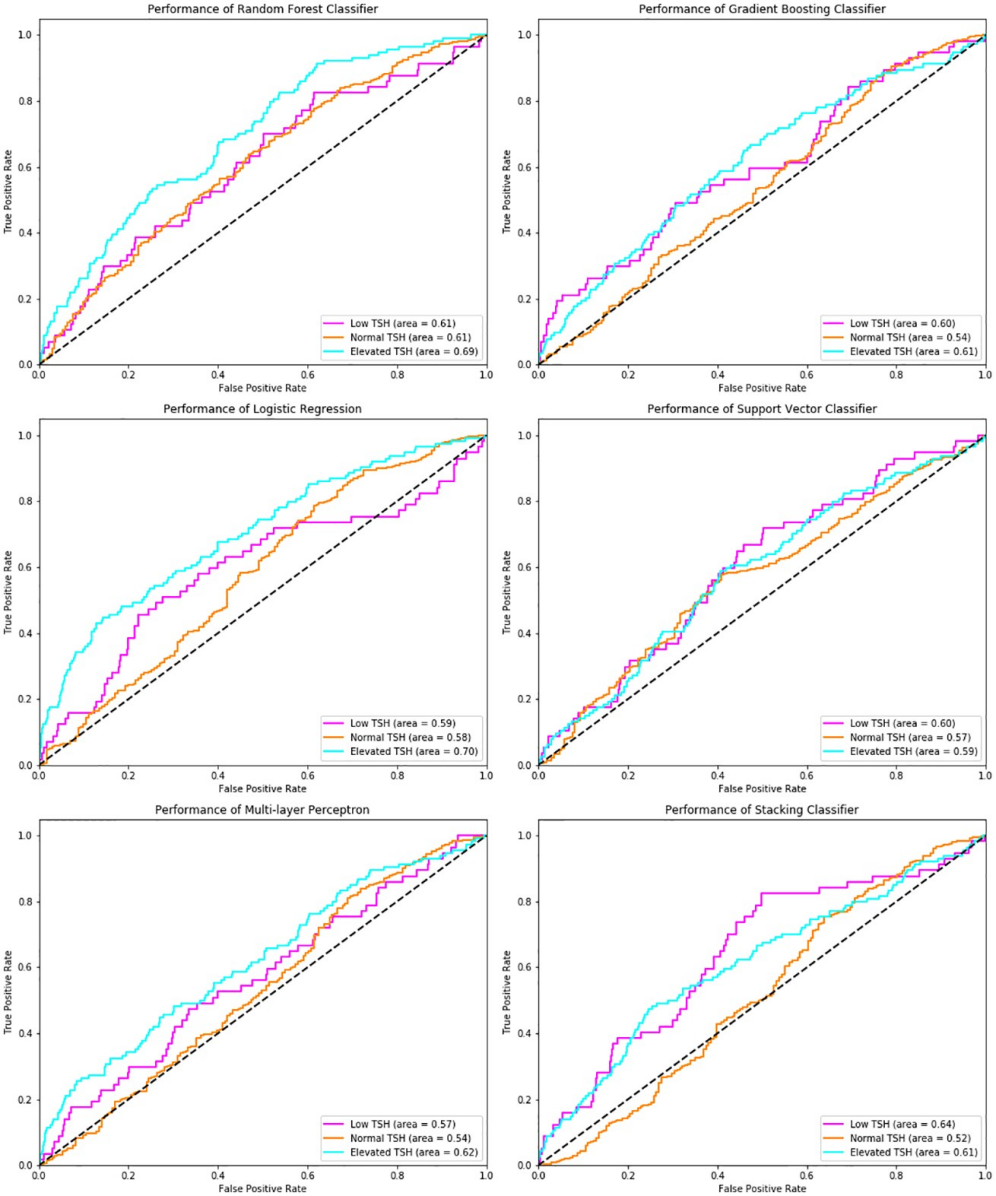
ROC curves—This shows the Receiver Operating Characteristic (ROC) curves which are used to evaluate the performance of various classification algorithms in a testing dataset. The ROC curve is plotted for every class i.e. low, normal and elevated. This figure also shows the Area Under ROC Curve (AUROC) for each class.

**Fig 5:**
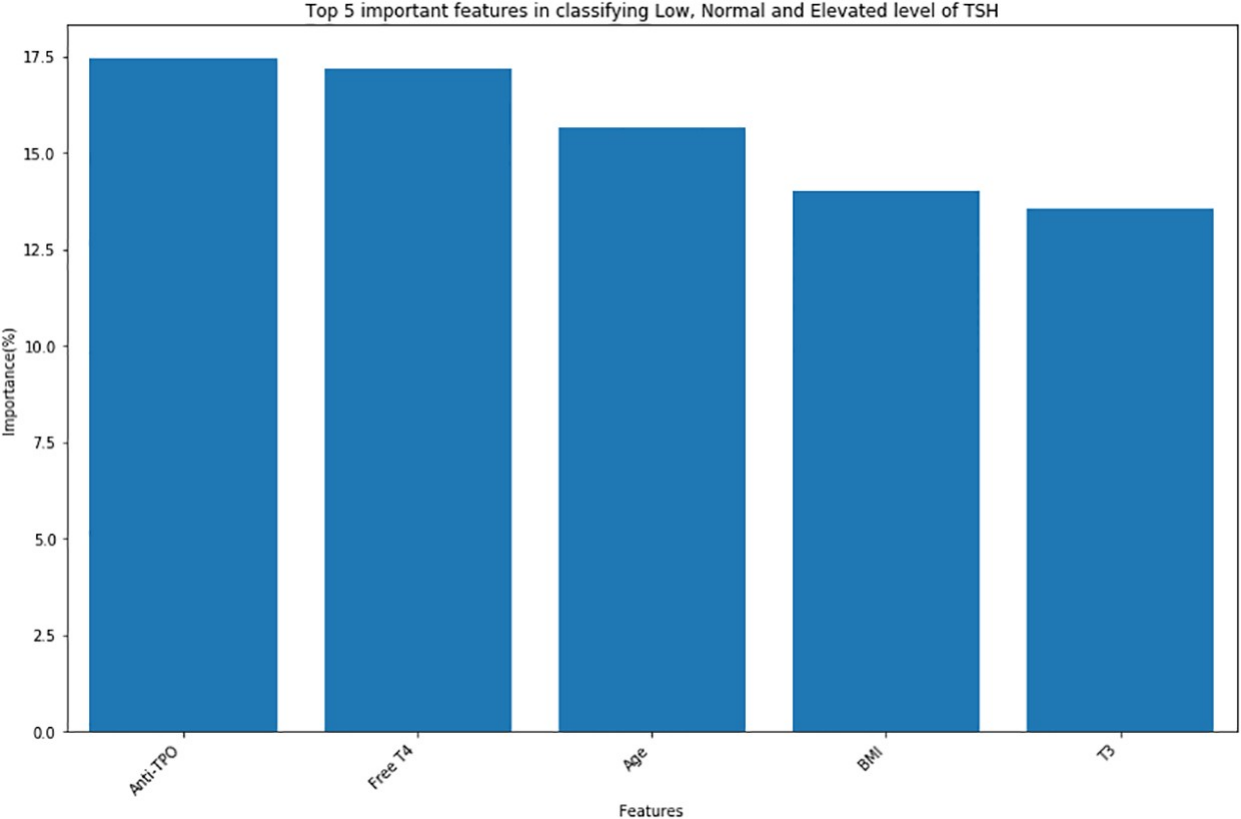
Feature importance for classification models—This shows the top 5 important features for classifying low, normal and elevated level of TSH using random forest in a testing dataset.

“Fig 5” shows that Anti-TPO is the most important feature in classifying TSH followed by Free-T4, Age, BMI and T3.

We also computed the precision, recall and F1-measure to compare the performance of different machine learning models. Table 6 below shows the performance of the different machine learning classification models.

**Table 6:** Performance of classification models (testing data).

Model	Low TSH			Normal TSH			Elevated TSH		
	Precision	Recall	F1	Precision	Recall	F1	Precision	Recall	F1
Random forest	0.04	0.05	0.04	0.95	0.91	0.93	0.10	0.18	0.13
Gradient boosting	0.11	0.07	0.09	0.94	0.97	0.96	0.12	0.07	0.09
Logistic regression	0.03	0.28	0.05	0.95	0.51	0.66	0.07	0.54	0.12
Support vector Classifier	0.03	0.09	0.04	0.95	0.87	0.91	0.06	0.12	0.08
Multi-layer perceptron	0.03	0.18	0.05	0.95	0.71	0.81	0.07	0.37	0.12
Stacking Classifier	0.12	0.05	0.07	0.94	0.98	0.96	0.11	0.05	0.07

(values in decimals mean %, for e.g. 0.95 implies 95%).

The classification metrices indicate that Random forest classifier performed the best in classifying elevated level of TSH.

## Discussion

Our study shows that, of the different machine learning methods used to analyse TSH (both as a continuous variable in regression analysis as well as a categorical variable for the classification studies) from commonly measured laboratory data and demographic variables, random forest performs well compared to other methods. Gradient Boosting and Stacking Regression also perform well in predicting TSH as a continuous variable. There was a reasonably high congruity between predicted and actual TSH.

To our knowledge, this is a first of a nature machine learning study on the epidemiological data that included laboratory and commonly obtained demographic information, for assessment of TSH values. Traditional statistical methods have been mostly challenging, in determining the complex relationship between thyroid hormones (triiodothyronine, free thyroxine), age, BMI and ethnicity [19, 20]. Prior studies have shown that BMI and weight appear to influence changes in free T4 levels but smoking at the time of free T4 measurements appears to negate that influence [21]. Some other variables, like diurnal variations in TSH due to sleep patterns, that have been shown previously to affect TSH levels, cannot be assessed in cross-sectional data[22]. Machine learning methods, when applied to prospective longitudinal data substantially improve our understanding of the factors behind measured actual TSH level(s).

TSH has a wide range of measurement between 0.4–4.5(depending on the specific lab) but individual TSH set point maybe genetically predetermined [23, 24]. A substantial proportion of persons with hypothyroidism have persistent symptoms despite achieving “target” TSH levels[25]. Different approaches have been tried in the past including treatment with liothyronine (T3) for persons with persistent symptoms[1]. Normal TSH level might not be the same as ‘optimum’ TSH level for alleviation of signs and symptoms of hypothyroidism and there is lack of data with respect to targeting the right TSH for the individual patient based on different variables. Once hypothyroidism develops, the baseline TSH of the patient prior to the disease may never be known.

Artificial intelligence (AI) (including different machine learning methods) offers us an insight into the individualized target TSH while treating hyper and hypothyroidism. The information generated by AI might help in identifying near-optimal TSH levels. Identifying the ‘optimal’ TSH levels might help in personalizing the target TSH, especially when the baseline TSH of a person who has developed hypothyroidism later in life, might be unknown due to lack of a previous measurement (i.e. when he/she had been euthyroid with normal TSH levels). The findings could then be used to target the appropriate TSH by adjusting the levothyroxine dose.

There are limitations to our study and the machine learning methods in general. The study is cross-sectional and thyroid tests might not represent changing or dynamic values. The study might not incorporate all the available participant data (especially the TSH values), since the NHANES began collecting population-based data in the 90s. Also, there might be a larger set of population with thyroid problems that might not have been captured through questionnaires that are themselves subject to recall and bias. However, the overall sample is large enough to make meaningful predictions from Machine Learning methods. Machine learning does not often offer a quantified relationship like a generalized or general linear/multinomial regression. We have not included smoking in analysis, though it has been shown to affect TSH level in certain studies. Smoking has been shown to be associated with a low normal TSH and is negatively associated with serological evidence of thyroid autoimmunity [26]. However, even without smoking as a variable, a prediction value of the TSH may help us determine the approximate TSH in an individual who shares certain age, gender, anthropometric and demographic features.

## Conclusion

In summary, the study is a demonstration of the capabilities of AI in the field of population based thyroid research when compared to the traditional methods. Though the study has some limitations (lack of cross validation with another large-scale dataset), it offers a good insight into the factors determining TSH levels. AI and machine learning methods offer an insight into the complex hypothalamic-pituitary -thyroid axis and may help us in making appropriate therapeutic decisions (thyroid hormone dosing) for the individual patient. Further studies are needed in this direction.

## Supporting information

S1 Data (XLSX)

S1 File (IPYNB)
